# Quantification of *Plasmodium* ex vivo drug susceptibility by flow cytometry

**DOI:** 10.1186/s12936-015-0940-8

**Published:** 2015-10-24

**Authors:** Grennady Wirjanata, Irene Handayuni, Pak Prayoga, Dwi Apriyanti, Ferryanto Chalfein, Boni F. Sebayang, Steven Kho, Rintis Noviyanti, Enny Kenangalem, Brice Campo, Jeanne Rini Poespoprodjo, Ric N. Price, Jutta Marfurt

**Affiliations:** Global and Tropical Health Division, Menzies School of Health Research, Charles Darwin University, PO Box 41096, Casuarina, 0811 Darwin, Australia; Papuan Health and Community Development Foundation (PHCDF), Timika, Papua Indonesia; Eijkman Institute for Molecular Biology, Jl. Diponegoro 69, Jakarta, 10430 Indonesia; District Health Authority, Timika, Papua Indonesia; Medicines for Malaria Venture (MMV), 20 rte de Pré-Bois, PO Box 1826, 1215 Geneva 15, Switzerland; Department of Paediatrics, Faculty of Medicine, Gadjah Mada University, Yogyakarta, Indonesia; Nuffield Department of Clinical Medicine, Centre for Tropical Medicine and Global Health, University of Oxford, Oxford, UK

**Keywords:** Malaria, *Plasmodium falciparum*, *Plasmodium vivax*, Ex vivo drug susceptibility, Drug resistance, Flow cytometry

## Abstract

**Background:**

The emergence and spread of multidrug-resistant *Plasmodium falciparum* and *Plasmodium vivax* highlights the need for objective measures of ex vivo drug susceptibility. Flow cytometry (FC) has potential to provide a robust and rapid quantification of ex vivo parasite growth.

**Methods:**

Field isolates from Papua, Indonesia, underwent ex vivo drug susceptibility testing against chloroquine, amodiaquine, piperaquine, mefloquine, and artesunate. A single nucleic acid stain (i.e., hydroethidine (HE) for *P. falciparum* and SYBR Green I (SG) for *P. vivax*) was used to quantify infected red blood cells by FC-based signal detection. Data derived by FC were compared to standard quantification by light microscopy (LM). A subset of isolates was used to compare single and double staining techniques.

**Results:**

In total, 57 *P. falciparum* and 23 *P. vivax* field isolates were collected for ex vivo drug susceptibility testing. Reliable paired data between LM and FC was obtained for 88 % (295/334) of these assays. The median difference of derived IC_50_ values varied from −5.4 to 6.1 nM, associated with 0.83–1.23 fold change in IC_50_ values between LM and FC. In 15 assays (5.1 %), the derived difference of IC_50_ estimates was beyond the 95 % limits of agreement; in eleven assays (3.7 %), this was attributable to low parasite growth (final schizont count < 40 %), and in four assays (1.4 %) due to low initial parasitaemia at the start of assay (<2000 µl^−1^). In a subset of seven samples, LM, single and double staining FC techniques generated similar IC_50_ values.

**Conclusions:**

A single staining FC-based assay using a portable cytometer provides a simple, fast and versatile platform for field surveillance of ex vivo drug susceptibility in clinical *P. falciparum* and *P. vivax* isolates.

## Background

Drug resistance remains a major obstacle to malaria control and emphasizes the importance of maintaining surveillance of anti-malarial efficacy to ensure optimal patient management and timely revision of treatment guidelines. Clinical trials are logistically difficult to conduct and their interpretation is confounded by host, parasite and drug factors. In vitro drug susceptibility testing provides an useful tool for monitoring drug resistant malaria of the individual components of combination therapies and investigating potential novel anti-malarial compounds prior to clinical use [1].

Several drug susceptibility assays have been developed for anti-malarial compounds with parasite growth quantified by enzyme-linked immunosorbent assay (ELISA) [2–4], fluorometry [5], or flow cytometry (FC) [6–8]. These assays have been applied to *Plasmodium falciparum* laboratory and field isolates, but have been limited for *Plasmodium vivax* drug susceptibility testing which remains still mostly reliant on microscopic quantification of parasite maturation [9–13]. The inability to sustain *P. vivax* in in vitro culture results in drug testing having to be conducted on fresh isolates directly from patients with malaria; this is often undertaken in laboratories with limited resources. Quantification of parasite growth by light microscopy (LM) is relatively simple, inexpensive, and suitable for use in field settings. LM can also discriminate between different parasite stages, a feature that remains critical in quantifying short-term schizont maturation assays [10]. The marked stage-specificity of drug activity, particularly apparent for piperaquine in *P. falciparum* and for chloroquine in *P. vivax* assays, requires diligent attention to ensure a high proportion of early ring stages at the start of the assay [14]. However, LM has several significant shortcomings. The method requires skilled microscopists applying sustained concentration on a time-consuming task. Even when assays are performed by skilled microscopists, both inter-operator as well as intra-operator variation in parasite counts is observed, highlighting the subjective nature of the method [11]. LM is also unsuitable for medium to high throughput screening for novel drug candidates. Among the available drug susceptibility methods, FC-based approaches have the advantage of being able to identify different parasite stages and to deal with the low signal-to-noise ratio inherent with the low parasitaemia of clinical field isolates. Other colourimetric or fluorometric methods that depend on red blood cell lysis are vulnerable to auto-fluorescence which exacerbates the background noise [15, 16].

FC-based methods using a variety of staining and detection techniques have been developed and established for drug susceptibility testing in *P. falciparum* laboratory strains [17–19]. Although a simple, reagent-free assay based on the quantification of haemozoin, detected by the use of depolarizing side-scatter light filters has been reported [20, 21], most of the published assays are based on the detection of double-stranded DNA of *Plasmodium*-infected erythrocytes since, with the exception of reticulocytes which generally account for ≤1 % of the red blood cell mass, uninfected erythrocytes do not contain a nucleus [22]. Various nucleic acid dyes have been successfully applied to quantify parasite biomass, including Hoechst [8], SYBR Green I [18], Thiazole Orange [17], SYTO-16 [23], and hydroethidine [24]. To date, few studies have described FC-based approaches for measuring drug susceptibility in *P. falciparum* [25, 26] and *P. vivax* [27, 28] field isolates. The high capital and maintenance costs of the required hardware, the sensibility of its lasers, and the need for specifically trained personnel have also limited the applicability of the FC technology to field lab-based assays. However, the development of portable and affordable FC systems provides an excellent opportunity for facilitating and improving drug susceptibility testing in *Plasmodium* field isolates.

The application of FC-based methods to *P. vivax* isolates was first reported by Malleret et al. [29]. Russell and colleagues further modified this double staining method, demonstrating the feasibility of FC-based quantification of chloroquine and artesunate susceptibility in *P. falciparum* and *P. vivax* field isolates [27]. More recently, a similar approach using a combination of Hoechst 33342 and hydroethidine and a portable flow cytometer equipped with a near-UV laser has been described [28]. These studies showed good correlation between the LM- and FC-based methods. The aim of the current study was to rationalize the FC methods further by using a single stain technique that provides a simpler, more rapid and robust assay for higher throughput drug testing in the field.

## Methods

### Study site and subjects

The study was conducted at a field laboratory in Timika, Papua Province, Indonesia, a region where multidrug-resistant *P. falciparum* and CQ-resistant *P. vivax* are highly prevalent [10, 30, 31]. *Plasmodium* species isolates were collected between 2012 and 2015, from patients with malaria attending an outpatient clinic. Patients with symptomatic malaria were recruited into the study if they had a microscopically confirmed peripheral parasitaemia with monospecies of either *P. falciparum* or *P. vivax* between 2000 and 80,000 µL^−1^. Patients were excluded from the study when they were younger than 2 years of age, had a haemoglobin level below 7 g/dL, or had anti-malarial or antibiotic treatment during the previous month. After obtaining written informed consent, blood was collected by venepuncture. Host white blood cells (WBC) were removed by cellulose column filtration as previously described [32] and packed infected red blood cells (IRBC) were used for the ex vivo drug susceptibility assay.

### Ex vivo drug susceptibility assay

*Plasmodium* drug susceptibility was measured using a protocol modified from the World Health Organization (WHO) microtest as described previously [12, 13]. In brief, two hundred microlitres of a 2 % haematocrit blood medium mixture (BMM), consisting of RPMI 1640 medium plus 10 % matched human serum (*P. falciparum*) or McCoy’s 5A medium plus 20 % matched human serum (*P. vivax*), was added to each well of pre-dosed drug plates containing 11 serial concentrations (two-fold dilutions) of the anti-malarials (maximum concentration shown in parentheses) CQ (2992 nM), piperaquine (PIP, 1029 nM), mefloquine (MFQ, 338 nM), amodiaquine (AQ, 158 nM), and artesunate (AS, 49 nM). A candle jar was used to mature the parasites at 37 °C for 35–56 h. Incubation was stopped when >40 % of ring stage parasites had reached the mature schizont stage (i.e., ≥5 distinct nuclei per parasite) in the drug-free control well as determined by light microscopy (LM).

### Anti-malarial compounds

The anti-malarial drugs CQ, PIP, MFQ, AQ, and AS were obtained from the Worldwide Anti malarial Research Network (WWARN) QA/QC Reference Material Programme [33]. The drugs were prepared as 1 mg/mL stock solutions in distilled water (CQ, PIP, MFQ, and AQ) or 70 % ethanol (AS). Drug plates were pre-dosed by diluting the compounds in 50 % methanol, followed by lyophilization, and stored at 4 °C. All drugs tested were assayed in duplicates. Drug plates were quality controlled by measuring drug response profiles in the CQ-resistant and CQ-sensitive laboratory strains K1 and FC27, respectively, using the same method.

### *Plasmodium falciparum* culture strains

Laboratory strains K1 and FC27 were obtained from MR4 (BEI Resources, ATCC Manassas, Virginia, USA). The parasites were kept in continuous in vitro culture as described previously [34]. To obtain highly synchronous parasite cultures, sorbitol treatment was applied once every week as described elsewhere [35].

### Quantification of parasites by light microscopy

Thick blood films made from each well were stained with 5 % Giemsa solution for 30 min and examined microscopically. The number of mature schizonts per 200 asexual stage parasites was determined for each drug concentration and normalized to that of the control well.

### Quantification of parasites by flow cytometry

#### Staining of IRBCs for FC

The vital dye hydroethidine (HE, Sigma-Aldrich; excitation_max_/emission_max_ = 535/610 nm) was used to stain IRBCs for *P. falciparum* infections and the nucleic acid dye SYBR Green I (SG, Invitrogen-Molecular Probes; excitation_max_/emission_max_ = 497/520 nm) for *P. vivax* infections. HE was prepared as 10 mg/mL stock solution in dimethyl sulphoxide (DMSO) and stored at −20 °C. SG stock solution (10,000 × concentrate in DMSO) was stored as per the instruction manual at −20 °C. The working solution for HE was made by diluting the HE stock solution 1:250 in phosphate buffered saline (PBS, pH 7.4). The 1× working solution for SG was made by diluting the SG stock solution 1:10,000 in Tris-saline buffer (TSB, pH 8.5). Staining was performed by adding 50 µL HE or 50 µL SG working solution to pelleted IRBCs (i.e., centrifugation at 1500 rpm, for 2 min) from 50 µL of the 2 % haematocrit BMM (final HE and SG concentration: 0.63 mM and 1:2, respectively) for 25 min in the dark at 37 °C. After the incubation, IRBCs were washed (i.e., centrifugation at 1500 rpm, for 2 min) once with 200 μL PBS, followed by final re-suspension in 200 µL PBS.

To further validate the single stain method, comparison was made with the double staining method published previously [27] using a subset of isolates obtained within the whole study. For the double staining method, 20 µL of BMM from each well at harvest were transferred to an empty 96-well plate and stained with a mixture of 2 μL HE 10 mg/mL solution, 30 μL of SG 1× working solution, and 48 μL PBS (final HE and SG concentration: 0.63  mM and 1:3.3, respectively). The mixture was incubated for 20 min in the dark at room temperature. The reaction was stopped by adding 200 μL PBS to all wells.

#### Gating and quantification of parasites by FC

**Fig. 1:**
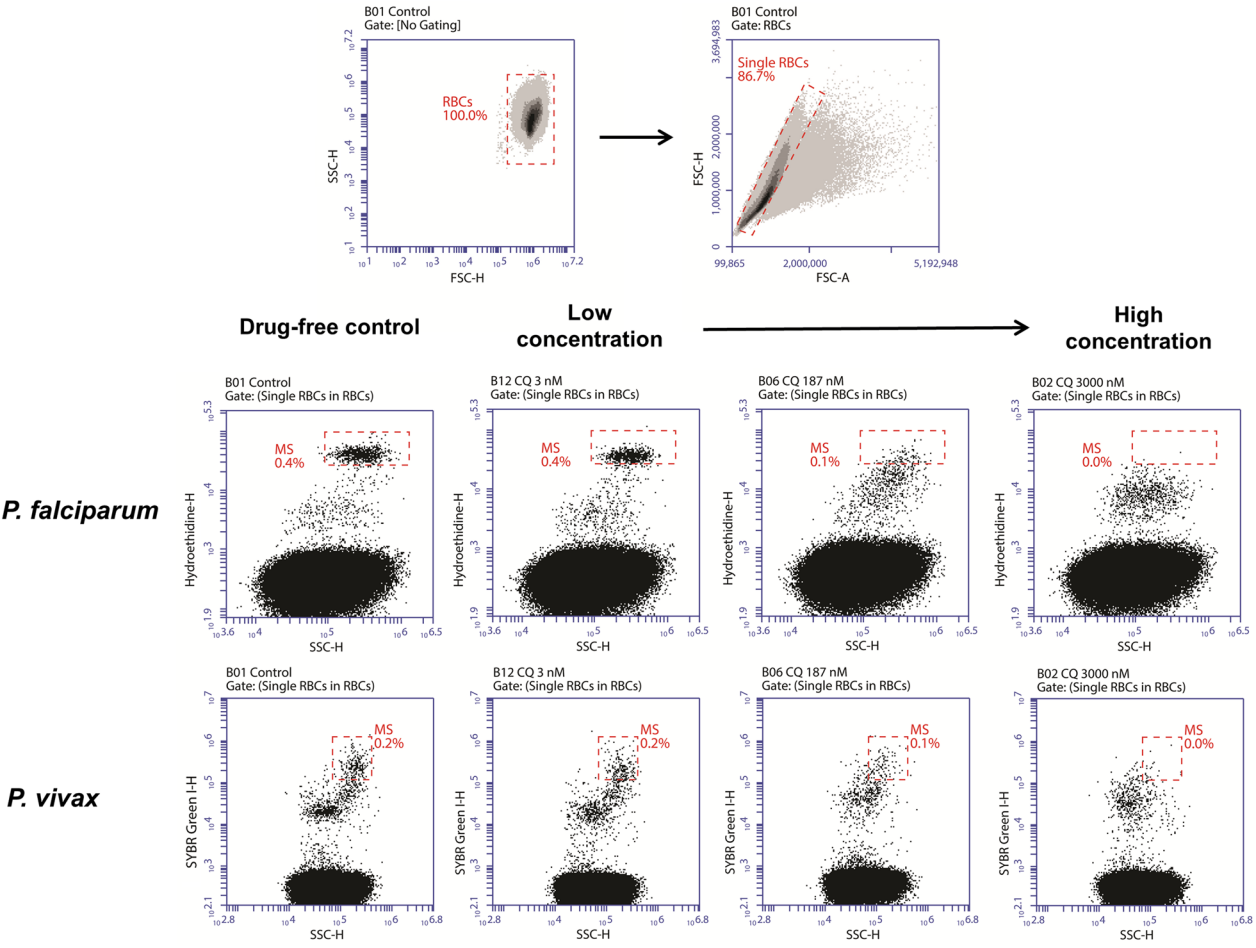
Gating strategy for mature schizonts. Red blood cells (RBCs) were identified and gated based on the forward/side scatter (FSC-H/SSC-H) dot plot in gate ‘RBCs’. The RBCs were visualized in a FSC-A/FSC-H dot plot to select cell singlets in gate ‘Single RBCs’. The single RBCs were then analysed in a SSC-H/Hydroethidine-H (FL2-H) dot plot for *P. falciparum* and a SSC-H/SYBR Green 1-H (FL1-H) dot plot for *P. vivax*. Mature schizonts were identified in gate ‘MS’ based on fluorescence intensity and side scatter values (i.e., increased DNA content and cell complexity) produced by schizonts compared to other *Plasmodium* life cycle stages

Samples were analysed using a dual-laser (blue: 488 nm, 50 mW solid state; red: 640 nm, 30 mW diode) 3/1 emission detection configuration BD Accuri C6™ cytometer system equipped with a high-throughput 96-well sampler (BD Accuri CSampler™). The blue laser was used for the detection of both dyes (SG in FL1: 530 ± 15 nm; HE in FL2: 585 ± 20 nm). The gating strategy is depicted in Fig. 1. First, cells were gated according to their FSC-H/SSC-H profile to exclude debris from the red blood cell (RBC) population. Gating was then applied on the FSC-A/FSC-H profile to differentiate cell doublets from single cells. The gated single cell population was analysed further in either the SSC-H/FL2-H (*P. falciparum*) or the SSC-H/FL1-H (*P. vivax*) dot plot profiles. The mature schizont gate was set based on the drug-free control well and applied to all drug-treated wells. Gating procedure for HE and SG double staining was applied as described by Russell et al. [27]. For data acquisition, 100,000 events were analysed. In the case of low parasitaemia (i.e., less than 0.2 %), the number of events analysed was increased to 200,000. Automated analysis was performed using the BD Accuri CFlow Sampler™ software.

### Statistical analysis

Dose–response data were analysed using nonlinear regression analysis (WinNonLn 4.1; Pharsight Corporation) and the 50 % inhibitory concentration (IC_50_) derived using an inhibitory sigmoid maximum effect (E_max_) model. IC_50_ ex vivo data were only accepted if the E_max_ and E_0_ of the predicted curve were within 15 % of 100 and 0, respectively. IC_50_ data with a high coefficient of variation (>100 %) and/or a high sum of squared residuals (>1) were also rejected.

Agreement between quantification methods was assessed by Bland–Altman analysis using log-transformed IC_50_ data obtained with both methods [36]. Wilcoxon signed-rank test or Student’s *t*-test on log-transformed data was used to assess the difference between paired samples using the different methods. All statistical analyses were carried out using Stata (version 13.1, College Station, Texas) and GraphPad Prism software (version 6.0).

### Ethics

Ethical approval for this project was obtained from the Human Research Ethics Committee of the Northern Territory Department of Health and Families and Menzies School of Health Research (HREC 2010-1396), Darwin, Australia, and the Eijkman Institute Research Ethics Commission (EIREC-47), Jakarta, Indonesia.

## Results

Between May 2012 and February 2015, 57 *P. falciparum* and 23 *P. vivax* isolates were collected and successfully harvested for the comparative analysis of flow cytometry (FC) and light microscopy (LM) based quantification. Baseline characteristics of the isolates are presented in Table 1.

**Table 1:** Baseline characteristics of isolates for which ex vivo assays were accomplished

Baseline characteristics	*P. falciparum* n = 73	*P. vivax* n = 28
Total number of isolates reaching harvest (%)	57 (78 %)	23 (82 %)
Median (range) delay from venepuncture to start of culture (minutes)	130 (80–225)	175 (90–330)
Median (range) duration of assay (hours)	44 (32–55)	46 (30–50)
Geometric mean (95 % CI) parasitaemia (asexual parasites/µL)	18,867 (14,423–24,680)	35,509 (20,433–61,707)
Median initial percentage (range) of parasites at ring stage	100^a^	96 (77–100)
Mean (95 % CI) schizont count at harvest^b^	45 (41–49)	40 (35–46)

*CI* confidence interval

^a^No range given (all values were 100 %)

^b^Percentage of mature schizonts per asexual blood stage parasites determined by light microscopy

Of the 334 drug assays attempted, reliable data could be derived from 329 (98.5 %) of those assessed by LM and 298 (89.2 %) by FC. The only difference in the proportion of successful assays arose for AS, for which LM produced reliable data in 96.5 % of *P. falciparum* isolates (55/57) and 100 % *P. vivax* isolates (23/23), compared to 66.7 % (38/57) and 73.9 % (17/23) assays by FC, respectively (Table 2). The low success rate for the artesunate assay was associated with the first batch of plates used in which 44.1 % (15/34) failed assays for both *P. falciparum* and *P. vivax*, whereas this failure rate fell to 15 % (7/44) in the second batch of plates tested (*p* = 0.004). For all of the other drugs tested, reliable data could not be obtained for a total of 10 assays and this was attributable to technical errors; for nine assays, the number of acquired events by FC was too low and for one assay, microscopy slide preparation was inaccurate.

**Table 2:** Percentage of successful assays with reliable data

Anti-malarial	*P. falciparum*			*P. vivax*		
	n^a^	Light microscopy	Flow cytometry	n^a^	Light microscopy	Flow cytometry
Chloroquine	57	56 (98.2 %)	55 (96.5 %)	23	23 (100 %)	21 (91.3 %)
Mefloquine	39	39 (100 %)	39 (100 %)	17	17 (100 %)	14 (82.3 %)
Piperaquine	57	56 (98.2 %)	55 (96.5 %)	23	23 (100 %)	21 (91.3 %)
Amodiaquine	25	24 (96.0 %)	25 (100 %)	13	13 (100 %)	13 (100 %)
Artesunate	57	55 (96.5 %)	38 (66.7 %)	23	23 (100 %)	17 (73.9 %)

^a^Number of drug assays attempted

**Fig. 2:**
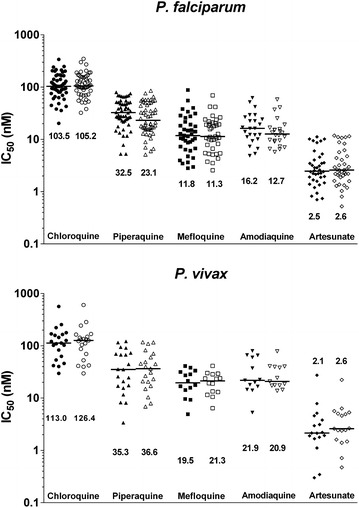
Ex vivo drug susceptibility of *P. falciparum* (upper pannel) and *P. vivax* (lower panel) clinical isolates by light microscopy (closed circles) or flow cytometry (open circles). *Numbers* represent median IC_50_s (nM) for each drug tested

In total, 294 (88 %) assays could be quantified by both LM and FC. The median IC_50_ values for all isolates with successful LM and FC assays are depicted in Fig. 2. These ex vivo drug susceptibility data are in concordance with ex vivo IC_50_ estimates produced in parallel studies in the area confirming a high level of CQ resistance in both *Plasmodium* species in Papua Indonesia [37, 38].

There were significant, albeit modest differences in IC_50_ values derived by LM and FC for PIP [median difference = 6.1 nM (range −18.7 to 21.0 nM)] and AQ [median difference = 2.8 nM (range −2.7 to 15.1 nM)], in *P. falciparum* isolates. No significant differences were observed between methodologies for *P. vivax* (Table 3).

**Fig. 3:**
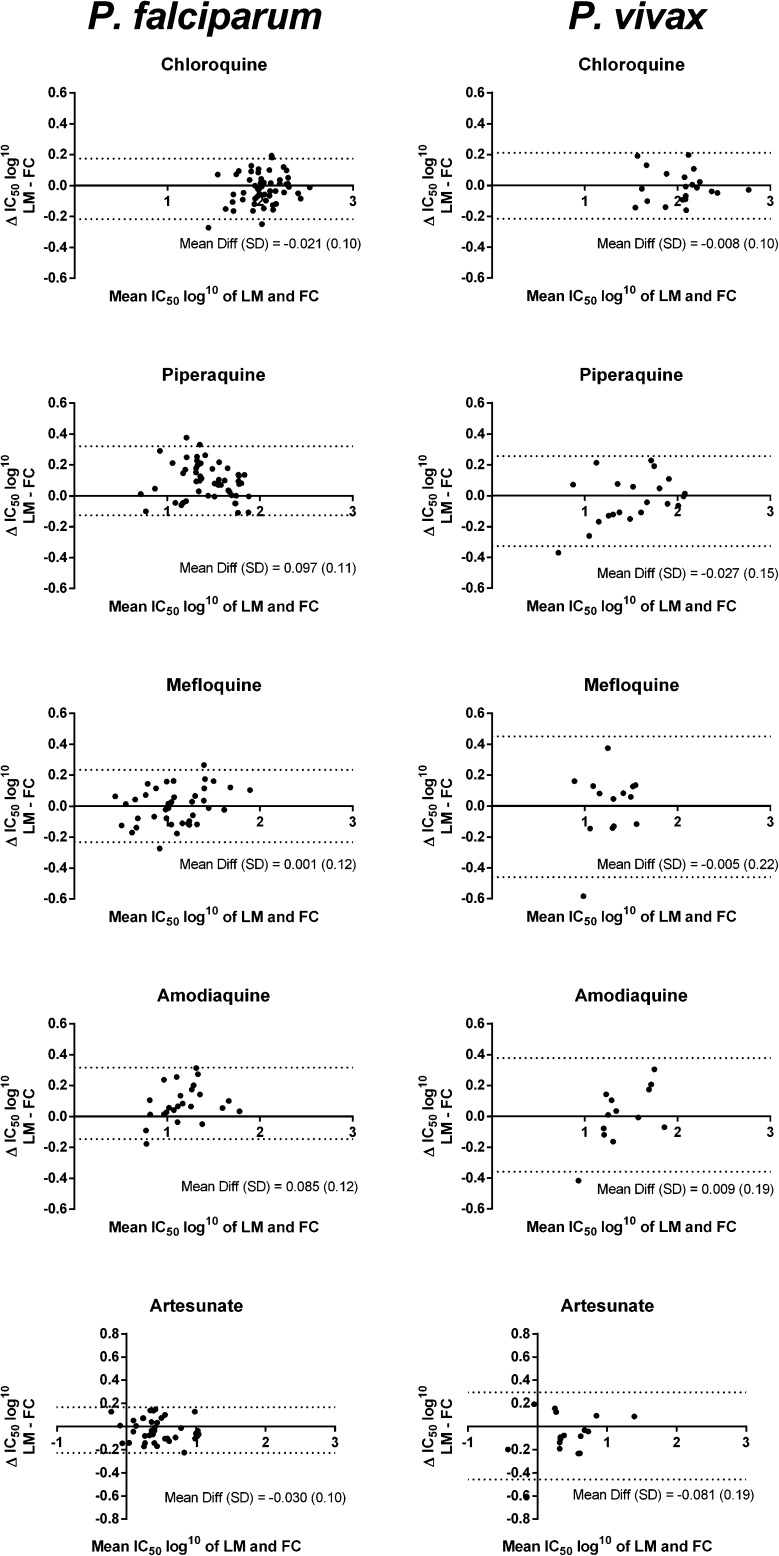
Bland-Altman plots of LM and FC derived IC_50_s. IC_50_ values of each drug tested in *P. falciparum* (left) and *P. vivax* (right). Dotted lines represent the 95 % limits of agreement between LM and FC

Bland–Altman plots are presented in Fig. 3 for each drug according to the species tested and the differences in IC_50_s in Table 3. From the 285 *P. falciparum* assays, there were 11 (3.86 %) outliers with differences in derived IC_50_s up to 60 nM for CQ, 18 nM for PIP, 19 nM for MFQ, 15 nM for AQ, and 3.6 nM for AS. In *P. vivax*, there were four (3.5 %) outliers out of 115 assays with maximum values of 60 nM for CQ, 27.5 nM for PIP, 40 nM for AQ, and 5 nM for AS. Eleven (73 %) of these outliers were identified to be attributable to isolates with a low initial parasitaemia at the start of the assays (<2000 parasites/µL), and four isolates due to low schizont counts (<40 %) at harvest associated with arrested trophozoite development or gametocytogenesis, ultimately resulting in sub-optimal harvest.

**Table 3:** Difference in IC_50_ estimates (nM) derived by light microscopy (LM) and flow cytometry (FC)

Anti-malarial	*P. falciparum*				*P. vivax*			
	n	Mean [95 % CI] difference in IC_50_ LM-FC (nM)	Median [Range] difference in IC_50_ LM-FC (nM)	Δ IC_50_ LM-FC *p* value^a^	n	Mean [95 % CI] difference in IC_50_ LM-FC (nM)	Median [Range] difference in IC_50_ LM-FC (nM)	Δ IC_50_ LM-FC *p* value^a^
Chloroquine	54	−3.8 [−10.7 to 3.2]	−5.1 [−60.9–59.4]	0.124	21	−4.5 [−16.0 to 7.1]	−5.4 [−46.1–60.2]	0.725
Piperaquine	54	6.0 [3.8 to 8.2]	6.1 [−18.7–21.3]	<0.001	21	0.7 [−4.6 to 6.0]	−4.5 [−15.2–27.5]	0.425
Mefloquine	39	1.3 [−0.6 to 3.1]	0.1 [−5.7–18.6]	0.969	14	1.2 [−3.6 to 6.0]	2.8 [−13.9–15.8]	0.931
Amodiaquine	24	4.0 [1.9 to 6.0]	2.8 [−2.7–15.1]	0.002	13	4.7 [−4.3 to 13.7]	0.4 [−11.8–40.2]	0.864
Artesunate	38	−0.4 [−0.7 to −0.01]	−0.25 [−3.6–2.9]	0.077	17	−0.12 [−0.9 to 0.7]	−0.45 [−2.2–5.0]	0.102

*CI* confidence interval

^a^ *p*-values obtained from paired *t-* test on log-transformed IC_50_ derived by LM and FC

To compare single with double staining, a subset of seven isolates (5 *P. falciparum* and 2 *P. vivax*) were assessed by both the single staining method presented in the current study and the double staining method published by Russell et al. [27]. The results are summarized in Table 4. Overall, similar IC_50_ values were obtained when these methods were compared in both *P. falciparum* laboratory strains and clinical *P. falciparum* and *P. vivax* field isolates.

**Table 4:** IC_50_ values derived by different staining methods in *P. falciparum* laboratory strains and clinical *Plasmodium* isolates

Anti-malarial	Mean IC_50_ (nM) in *P. falciparum* laboratory lines^a^						Clinical *Plasmodium* isolates					
	FC27 (CQ sensitive)			K1 (CQ resistant)			*P. falciparum* (n = 5) Median IC_50_ (nM, range)			*P. vivax* (n = 2) IC_50_ estimates (nM)		
	Single^b^	Double^c^	LM^d^	Single^b^	Double^c^	LM^d^	Single^b^	Double^c^	LM	Single^e^	Double^c^	LM^d^
CQ	25.1	23.7	20.7	171.0	167.0	159.4	85.0 (52.8–165.0)	97.3 (52.9–179.0)	108 (65.6–115.0)	92.2; 132.0	88.5; 150.0	102.0; 113.0
PIP	32.8	32.5	34.7	63.0	63.9	89.7	35.0 (19.9–45.0)	38.6 (12.6–57.1)	47.5 (27.6–68.4)	79.1; 153.0	85.6; 155.0	91.8; 115.0
MFQ	46.8	40.9	49.9	16.6	14.5	9.4	10.2 (3.5–19.3)	10.2 (2.2–23.3)	9.4 (3.6–15.5)	23.0; 43.0	30.3; 42.4	17.7; 22.6
AS	4.1	4.3	2.6	8.7	7.8	6.0	2.2 (1.3–3.1)	2.2 (1.9–3.7)	1.9 (1.2–2.6)	2.6; 6.3	2.8; 6.7	3.1; 7.8

*CQ* chloroquine, *PIP* piperaquine, *MFQ* mefloquine, *AS* artesunate

^a^Mean IC_50_s (derived from 2 independent experiments)

^b^Assay quantification by flow cytometry (FC) using single staining with hydroethidine (HE) for *P. falciparum*

^c^Assay quantification by FC using double staining with HE and SG for both species [27, 29]

^d^ *LM* light microscopy

^e^Assay quantification by flow cytometry (FC) using single staining with SYBR Green I (SG) for *P. vivax*

## Discussion

Methods for quantifying ex vivo growth of *Plasmodium* that include parasite staging are currently limited to light microscopy (LM) and flow cytometry (FC). The present study provides a head-to-head comparison of these two approaches highlighting the utility and advantages of a single staining FC method. The assay was easy to apply in a remote field laboratory and produced results comparable to the LM-based method. Successful paired assay data could be generated in 88 % of drug assays, although the success rate was somewhat lower for AS when using FC (66.7 and 73.9 % for *P. falciparum* and *P. vivax*, respectively), compared to LM (96.5 and 100 % for *P. falciparum* and *P. vivax*, respectively). The failure to quantify artesunate susceptibility appeared to be related to a specific batch of drug plates that was, by mistake, stored at ambient temperature which resulted in 44.1 % (15/34) failed assays compared to a subsequent batch in which the number of failed assay decreased to 15 % (7/44).

During the initial phase of assay development using *P. falciparum* laboratory strains, cell staining was performed using HE. However, early experiments with *P. vivax* field isolates did not yield consistent results, with some isolates failing to produce sufficient fluorescent signals to distinguish both uninfected RBCs versus infected RBCs (IRBC) and the different life cycle stages; the results confounded the gating procedure for *P. vivax* isolates. Alternative nucleic acid (NA) dyes were tested (SYBR Safe nucleic acid stain, Thiazole Orange, SYTO 16, and SYBR Green I). SYBR Green I (SG) was chosen for *P. vivax* since it resulted in significantly better resolution of the different RBC and IRBC populations with clear separation between schizonts and other life cycle stages. However, the HE stain produced the optimal results for *P. falciparum* isolates, with good resolution of different RBC and IRBC populations and higher signal-to-noise ratio compared to SG. A comparative analysis of the single staining method with the HE plus SG double staining method previously published by Russell et al. [27] was conducted in two *P. falciparum* laboratory strains and a subset of five *P. falciparum* and two *P. vivax* clinical field isolates summarized in Table 4. Both methods produced comparable results and appear suitable for assessing anti-malarial drug susceptibility (Table 2). The advantage of using HE or SG single staining is that there is no need to run single stain controls in each experiment in order to compensate for spectral overlap that occurs when double staining methods using fluorescent dyes with close excitation/emission spectra such as HE and SG are applied. Therefore, the presented single staining method greatly facilitates raw FC data analysis.

Overall, the drug susceptibility data quantified by LM and FC were similar. Statistically significant differences in IC_50_ values were observed for PIP and AQ in *P. falciparum*. However, the median differences were modest (6.1 nM for PIP and 2.8 nM for AQ), all within the confidence bounds of the assay and therefore, unlikely to be of biological or clinical significance. In total, there were 15 outliers with higher differences in IC_50_s derived by FC and LM. Several factors are likely to have contributed to these outliers, the most notable of which was a reduced parasite count, either from a low starting parasitaemia (lower than 2000 parasites/µL), or inadequate parasite growth leading to a low final mature schizont count below 40 %. Quantification of low parasite densities is a recognized factor undermining the confidence of LM or FC [10, 14]. Given the time-consuming and subjective nature of LM-based assays, those assays with reduced parasite count increase the workload on the microscopists, which leads to lower assay reading accuracy. The reliability of FC data can be improved by increasing the number of acquired events that enables greater confidence in gating the schizont population. Another potential confounding factor in the comparison of the LM and FC data is the presence of abnormal (unhealthy) schizonts, which occur following drug treatment and/or sub-optimal growth. The LM-based method requires visual quantification of mature schizonts with >5 chromatin dots, whereas abnormal schizonts with irregular chromatin dots plus altered shape and size are not classified as mature schizonts. However, these abnormal schizonts cannot be readily distinguished with the FC method since quantification is based on side scatter properties and nucleic acid content, irrespective of the parasites’ fitness.

The FC-based quantification approach offers fast, field applicable, objective and transparent post assay sample processing and data acquisition. LM-based quantification requires assay harvest, thick blood film preparation from each drug well, slide drying, Giemsa staining, followed by slide reading and cross checking of microscopy. For a standard assay of one *Plasmodium* isolate, testing four drugs on a 96-well format, this can be accomplished between 16 and 20 h. The FC-based approach requires cell staining, washing, and final re-suspension and data acquisition by FC, with a total average processing time of 2 h. The assay procedure *per se* (i.e., sample preparation and assay duration of 52–56 h) and data analysis (i.e. pharmacodynamics modelling of raw drug response data) does not differ between the two approaches. Hence, between 14 and 18 man hours can be saved, varying according to the LM and FC skills of the operators.

The FC approach also has the advantage of requiring a smaller volume of blood than LM quantification. In a typical run, less than 0.2 µL packed RBCs from the 2 % haematocrit BMM is required for each assay. A reduction in the blood volume required for each drug assay should increase the number of drugs that can be tested in parallel from the same volume of blood. The savings in processing costs and human resources counter balance the higher initial costs for hardware and maintenance for FC and make this a viable field adapted technology.

## Conclusion

The single staining FC-based quantification of ex vivo parasite growth provides a rapid and reliable estimate of drug response. Its implementation in field laboratories to replace or complement the time-consuming and labour-intensive LM-based method has potential to greatly facilitate surveillance and screening of anti-malarial agents.

## Abbreviations

AQ: amodiaquine
AS: artesunate
BMM: blood medium mixture
CQ: chloroquine
ELISA: enzyme-linked immunosorbent assay
FC: flow cytometry
HE: hydroethidine
IC_50_: half-maximal inhibition of growth
IRBC: infected red blood cells
WBC: white blood cell
LM: light microscopy
MMV: Medicines for Malaria Venture
MFQ: mefloquine
PIP: piperaquine
RBC: red blood cells
SG: SYBR Green I
WHO: World Health Organization
WWARN: WorldWide Antimalarial Resistance Network

## References

[1] Desjardins RE, Canfield CJ, Haynes JD, Chulay JD (1979). Quantitative assessment of antimalarial activity in vitro by a semiautomated microdilution technique. Antimicrob Agents Chemother.

[2] Noedl H, Bronnert J, Yingyuen K, Attlmayr B, Kollaritsch H, Fukuda M (2005). Simple histidine-rich protein 2 double-site sandwich enzyme-linked immunosorbent assay for use in malaria drug sensitivity testing. Antimicrob Agents Chemother.

[3] Noedl H, Yingyuen K, Laoboonchai A, Fukuda M, Sirichaisinthop J, Miller RS (2006). Sensitivity and specificity of an antigen detection ELISA for malaria diagnosis. Am J Trop Med Hyg.

[4] Kaddouri H, Nakache S, Houzé S, Mentré F, Le Bras J (2006). Assessment of the drug susceptibility of Plasmodium falciparum clinical isolates from africa by using a Plasmodium lactate dehydrogenase immunodetection assay and an inhibitory maximum effect model for precise measurement of the 50-percent inhibitory concentration. Antimicrob Agents Chemother.

[5] Smilkstein M, Sriwilaijaroen N, Kelly JX, Wilairat P, Riscoe M (2004). Simple and inexpensive fluorescence-based technique for high-throughput antimalarial drug screening. Antimicrob Agents Chemother.

[6] Van Vianen PH, Thaithong S, Reinders PP, Van Engen A, Van Der Keur M, Tanke HJ (1990). Automated flow cytometric analysis of drug susceptibility of malaria parasites. Am J Trop Med Hyg.

[7] Pattanapanyasat K, Thaithong S, Kyle DE, Udomsangpetch R, Yongvanitchit K, Hider RC, Webster HK (1997). Flow cytometric assessment of hydroxypyridinone iron chelators on in vitro growth of drug-resistant malaria. Cytometry.

[8] Grimberg BT, Erickson JJ, Sramkoski RM, Jacobberger JW, Zimmerman PA (2008). Monitoring Plasmodium falciparum growth and development by UV flow cytometry using an optimized Hoechst thiazole orange staining strategy. Cytometry A.

[9] Russell BM, Udomsangpetch R, Rieckmann KH, Kotecka BM, Coleman RE, Sattabongkot J (2003). Simple in vitro assay for determining the sensitivity of Plasmodium vivax isolates from fresh human blood to antimalarials in areas where P. vivax is endemic. Antimicrob Agents Chemother.

[10] Russell B, Chalfein F, Prasetyorini B, Kenangalem E, Piera K, Suwanarusk R (2008). Determinants of in vitro drug susceptibility testing of Plasmodium vivax. Antimicrob Agents Chemother.

[11] Russell B, Suwanarusk R, Malleret B, Costa FT, Snounou G, Baird JK (2012). Human ex vivo studies on asexual Plasmodium vivax: the best way forward. Int J Parasitol.

[12] Marfurt J, Chalfein F, Prayoga P, Wabiser F, Wirjanata G, Sebayang B (2012). Comparative ex vivo activity of novel endoperoxides in multidrug-resistant plasmodium falciparum and P. vivax. Antimicrob Agents Chemother.

[13] Marfurt J, Chalfein F, Prayoga P, Wabiser F, Kenangalem E, Piera KA (2011). Ex vivo drug susceptibility of ferroquine against chloroquine-resistant isolates of Plasmodium falciparum and P. vivax. Antimicrob Agents Chemother.

[14] Kerlin DH, Boyce K, Marfurt J, Simpson JA, Kenangalem E, Cheng Q (2012). An analytical method for assessing stage-specific drug activity in Plasmodium vivax malaria: implications for ex vivo drug susceptibility testing. PLoS Neglect Trop Dis.

[15] Druilhe P, Brasseur P, Blanc C, Makler M (2007). Improved assessment of Plasmodium vivax response to antimalarial drugs by a colorimetric double-site plasmodium lactate dehydrogenase antigen capture enzyme-linked immunosorbent assay. Antimicrob Agents Chemother.

[16] Kosaisavee V, Suwanarusk R, Nosten F, Kyle DE, Barrends M, Jones J (2006). Plasmodium vivax: isotopic, PicoGreen, and microscopic assays for measuring chloroquine sensitivity in fresh and cryopreserved isolates. Exp Parasitol.

[17] Makler MT, Lee LG, Recktenwald D (1987). Thiazole orange: a new dye for Plasmodium species analysis. Cytometry.

[18] Izumiyama S, Omura M, Takasaki T, Ohmae H, Asahi H (2009). Plasmodium falciparum: development and validation of a measure of intraerythrocytic growth using SYBR Green I in a flow cytometer. Exp Parasitol.

[19] Jouin H, Daher W, Khalife J, Ricard I, Puijalon OM, Capron M, Dive D (2004). Double staining of Plasmodium falciparum nucleic acids with hydroethidine and thiazole orange for cell cycle stage analysis by flow cytometry. Cytometry A.

[20] Rebelo M, Sousa C, Shapiro HM, Mota MM, Grobusch MP, Hanscheid T (2013). A novel flow cytometric hemozoin detection assay for real-time sensitivity testing of Plasmodium falciparum. PLoS One.

[21] Rebelo M, Tempera C, Fernandes JF, Grobusch MP, Hanscheid T (2015). Assessing anti-malarial drug effects ex vivo using the haemozoin detection assay. Malar J.

[22] Grimberg BT (2011). Methodology and application of flow cytometry for investigation of human malaria parasites. J Immunol Meth.

[23] Jiménez-Díaz MB, Mulet T, Gomez V, Viera S, Alvarez A, Garuti H (2009). Quantitative measurement of Plasmodium-infected erythrocytes in murine models of malaria by flow cytometry using bidimensional assessment of SYTO-16 fluorescence. Cytometry A.

[24] Van der Heyde HC, Elloso MM (1995). Use of hydroethidine and flow cytometry to assess the effects of leukocytes on the malarial parasite Plasmodium falciparum. Clin Diagn Lab Immunol.

[25] Karl S, Wong RP, St Pierre TG, Davis TM (2009). A comparative study of a flow-cytometry-based assessment of in vitro Plasmodium falciparum drug sensitivity. Malar J.

[26] Woodrow CJ, Wangsing C, Sriprawat K, Christensen P, Nosten F, Rénia L (2015). A comparison between flow cytometry, microscopy and lactate dehydrogenase ELISA for Plasmodium falciparum drug-susceptibility testing under field conditions. J Clin Microbiol.

[27] Russell B, Malleret B, Suwanarusk R, Anthony C, Kanlaya S, Lau Y (2013). Field-based flow cytometry for ex vivo characterization of Plasmodium vivax and P. falciparum antimalarial sensitivity. Antimicrob Agents Chemother.

[28] Suwanarusk R, Russell B, Ong A, Sriprawat K, Chu CS, PyaePhyo A, et al. Methylene blue inhibits the asexual development of vivax malaria parasites from a region of increasing chloroquine resistance. J Antimicrobial Chemother. 2014; dku326. 10.1093/jac/dku326 PMC4267499 25150147

[29] Malleret B, Claser C, Ong ASM, Suwanarusk R, Sriprawat K, Howland SW (2011). A rapid and robust tri-color flow cytometry assay for monitoring malaria parasite development. Sci Rep.

[30] Karyana M, Burdarm L, Yeung S, Kenangalem E, Wariker N, Maristela R (2008). Malaria morbidity in Papua Indonesia, an area with multidrug resistant Plasmodium vivax and Plasmodium falciparum. Malar J.

[31] Ratcliff A, Siswantoro H, Kenangalem E, Wuwung M, Brockman A, Edstein MD (2007). Therapeutic response of multidrug-resistant Plasmodium falciparum and P. vivax to chloroquine and sulfadoxine-pyrimethamine in southern Papua, Indonesia. Trans R Soc Trop Med Hyg.

[32] Venkatesan M, Amaratunga C, Campino S, Auburn S, Koch O, Lim P (2012). Using CF11 cellulose columns to inexpensively and effectively remove human DNA from Plasmodium falciparum-infected whole blood samples. Malar J.

[33] Lourens C, Watkins WM, Barnes KI, Sibley CH, Guerin PJ, White NJ (2010). Implementation of a reference standard and proficiency testing programme by the World Wide Antimalarial Resistance Network (WWARN). Malar J.

[34] Trager W, Jensen JB (1976). Human malaria parasites in continuous culture. Science.

[35] Lambros C, Vanderberg JP (1979). Synchronization of Plasmodium falciparum erythrocytic stages in culture. J Parasitol.

[36] Bland JM, Altman DG (1986). Statistical methods for assessing agreement between two methods of clinical measurement. Lancet.

[37] Wirjanata G, Sebayang BF, Chalfein F, Prayoga, Handayuni I, Noviyanti R (2015). Contrasting ex vivo efficacies of “reversed chloroquine” compounds in chloroquine-resistant Plasmodium falciparum and P. vivax isolates. Antimicrob Agents Chemother.

[38] Wirjanata G, Sebayang BF, Chalfein F, Prayoga, Handayuni I, Trianty L (2015). Potent ex vivo activity of naphthoquine and methylene blue against drug-resistant clinical isolates of Plasmodium falciparum and Plasmodium vivax. Antimicrob Agents Chemother.

